# The Efficacy of Composite Essential Oils against Aflatoxigenic Fungus *Aspergillus flavus* in Maize

**DOI:** 10.3390/toxins12090562

**Published:** 2020-09-01

**Authors:** Fangzhi Xiang, Qianqian Zhao, Kai Zhao, Hao Pei, Fang Tao

**Affiliations:** School of Life Sciences, Anhui Agricultural University, Hefei 230036, China; xiangfangzhi@ahau.edu.cn (F.X.); zhaoqianqian@ahau.edu.cn (Q.Z.); zhaokai@ahau.edu.cn (K.Z.); peihao@ahau.edu.cn (H.P.)

**Keywords:** *Aspergillus flavus*, composite essential oils, synergistic effect, aflatoxin B1, maize grains

## Abstract

The efficacy of eleven essential oils (EOs) against *Aspergillus flavus* NRRL 3357 was investigated. The highest antifungal activity against this aflatoxigenic fungus was exhibited by cinnamon, oregano and lemongrass, which showed low minimum inhibitory concentration (MIC) values under vapor conditions. Interactions of the three EOs were evaluated by the fractional inhibition concentration index (FICI), and the composite essential oils (CEO) showed synergistic inhibitory activities. Chemical analysis of the composite essential oils of cinnamon, oregano, and lemongrass (COL-CEO) revealed that (Z)-citral (33.44%), (E)-citral (32.88%) and carvacrol (19.84%) were the dominant components, followed by limonene (4.29%) and cinnamaldehyde (3.76%). COL-CEO not only inhibited fungal growth but also decreased aflatoxin B1 production by *A. flavus*. Downregulation of the relative expression of aflatoxin genes in the aflatoxin biosynthetic pathway by COL-CEO revealed its anti-aflatoxigenic mechanism. COL-CEO could also affect the colonization of *A. flavus* on maize grains. Therefore, COL-CEO may be considered as a potential natural antifungal agent, which could be used for the storage of maize and other grains.

## 1. Introduction

Cereals can be contaminated by molds in the field or during storage [1], and are prone to mildew attacks, especially under high temperature and precipitation conditions [2,3,4]. Food spoilage caused by toxigenic fungi has increasingly been a severe problem. A large number of food crops all over the world are contaminated with mycotoxins every year [5]. Cereals contaminated with mycotoxins are not only harmful to animal and human health, but also cause substantial economic losses [6]. The most common toxigenic fungi in animal feed are *Alternaria*, *Aspergillus*, *Fusarium* and *Penicillium* [7,8,9]. Of these, fungi within *Aspergillus* section *Flavi*, particularly *A. flavus*, have received the most attention, as they include the most frequently detected species that produces aflatoxin B1 (AFB1) [10,11]. AFB1 is the most hepatotoxic and carcinogenic secondary metabolite, and has been classified as a Group I carcinogen to humans by the International Agency for Research (IARC) [12]. Even low doses of aflatoxin B1 pose chronic health risks, whereas higher dosages can be lethal [13,14]. Contaminated cereals, such as maize, rice, sorghum and wheat, nuts and spices are potential sources of exposure to aflatoxins [15,16,17]. In particular, maize, which is the primary source of nutrition in several parts of the world, is highly susceptible to aflatoxin contamination [18]. Maximum levels (MLs) of AFB1 in food crops for human consumption have been legislated in many countries. For example, in the USA, MLs for AFB1 in all food crops are 20 μg/kg [19]. Chinese legislation establishes different MLs for AFB1 in various foods, i.e., 20, 10 and 5 μg/kg for maize, rice and wheat products, respectively. The ML for AFB1 in the European Union for rice and maize is 5 μg/kg, whereas in other cereals, it is regulated at 2 μg/kg [5]. Trade barriers have caused significant economic losses to countries due to differences in legislated standards. Owing to the health and economic repercussions of aflatoxin contaminated food and feed, it is necessary to regulate aflatoxin levels in food and feed, which may be achieved by inhibiting the growth and proliferation of *A. flavus*.

Synthetic chemicals such as propionic acid [20,21] and phosphine [22] are used as fungicidal preservatives in grain storage. Moreover, chemicals such as coumarines [23], tiadiazines [24], fullerol [25,26] and fullerene [27] can inhibit the growth of *A. flavus* and the production of aflatoxins. Although chemical preservatives can inhibit the growth of mold and ensure food safety, the potential undesirable effects on the human body and environmental problems may limit their usage [28]. In recent years, natural antifungal agents with low toxicity have received considerable attention. For instance, essential oils from plants and marine organisms [29] and a variety of bioactive compounds such as aldehydes, phenols and alcohols have significant bioactivities against fungal growth. Moreover, cinnamon [30], oregano [31], lemongrass [32], clove [33], basil [34] and thyme essential oils (EOs) [35] have been shown to effectively inhibit fungal growth and toxin synthesis. For instance, the antifungal activity of basil EO can be 64–67% higher than that of the standard fungicide carbendazim against the two fungal species that commonly occur in food, *Penicillium chrysogenum* and *Aspergillus flavus* [36]. Similarly, clove EO and vatica EO have inhibitory effects on spore germination and mycelial growth of *A. flavus* [37]. Moreover, composite essential oils (CEOs) showed better inhibitory effects than single essential oils, because of the synergistic effects of various components in the composite essential oils [38,39,40]. Although the inhibitory effect of CEO has been evaluated against *Fusarium* pathogens and fumonisin production, the same has not been tested extensively against *A. flavus* and aflatoxin production [41].

This study aimed to find a synergistic antifungal compound against *A. flavus* and demonstrate the antifungal effects of CEO on stored grains. In this study, the antifungal activities of eleven plant essential oils against aflatoxigenic wild-type *A. flavus* NRRL 3357 were tested via the vapor method, and the synergistic effect of three EOs with strong antifungal activities were tested. The chemical composition of CEO against aflatoxin production by *A. flavus* was also investigated. To elucidate the inhibitory mechanism of CEO on aflatoxin production, we evaluated the expression of several clustered genes in the aflatoxin biosynthetic pathway. Furthermore, the effect of CEO on contaminated maize kernels was also tested.

## 2. Results

### 2.1. Antifungal Effect of Essential Oils on Aspergillus flavus

**Figure 1:**
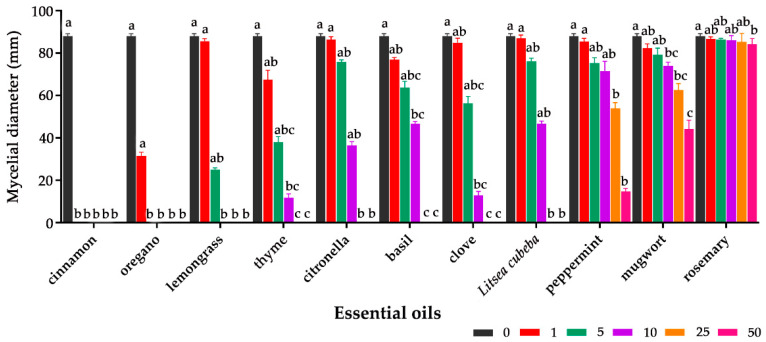
*Aspergillus flavus* mycelial growth at different concentrations (0, 1, 5, 10, 25 and 50 μL/disc) of eleven essential oils (cinnamon, oregano, lemongrass, clove, citronella, basil, thyme, *Litsea cubeba*, peppermint, mugwort and rosemary). Each value is the mean of three replicates (*n* = 3) ± standard deviation (SD). Kruskal–Wallis test was applied for non-parametric analysis of variance, and means for each concentration were separated by uncorrected Dunn’s test. Values with different lower-case letters were significantly different (*p* < 0.05).

The antifungal effect of eleven essential oils (EOs) on *A. flavus* was determined by measuring the mycelial diameter on potato dextrose agar (PDA) plates. The results of the vapor test showed that cinnamon essential oil was the most effective, as it completely inhibited the growth of *A. flavus* mycelia at 1 μL/disc. Oregano and lemongrass EOs were less effective, with no mycelial growth observed at 5 μL/disc and 10 μL/disc, respectively. Five EOs—basil, clove, citronella, *Litsea cubeba* and thyme—showed weaker inhibition. Mugwort, peppermint and rosemary EOs had the weakest inhibitory effect against *A. flavus* and were effective only at 50 μL/disc (Figure 1).

### 2.2. Determination of Minimum Inhibitory Concentration (MIC) of Cinnamon, Oregano and Lemongrass EOs

**Figure 2:**
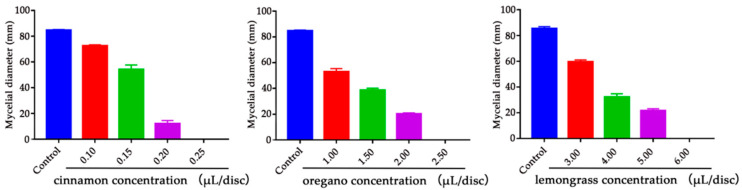
The MIC values of cinnamon, oregano, and lemongrass EOs against *A. flavus*. Error bars indicate mean (*n* = 3) ± standard deviation (SD). EO: essential oil, MIC: minimum inhibitory concentration.

Cinnamon, oregano and lemongrass EOs showed remarkable antifungal activity against *A. flavus*, as determined by MIC quantification. The MIC values of cinnamon, oregano and lemongrass EO vapors against *A. flavus* on PDA for seven days are shown in Figure 2. Different concentrations of the three EOs, i.e., cinnamon (0.25 μL/disc), oregano (2.50 μL/disc) and lemongrass (6.00 μL/disc), were found to completely inhibit fungal growth. This work further confirmed that cinnamon essential oil is the best antifungal agent among all tested EOs.

### 2.3. Combinations of EOs with Synergistic Inhibitory Activity against A. flavus

Owing to their superior antifungal activities, the combined antifungal effect of cinnamon, oregano and lemongrass EOs against *A. flavus* was tested. The results of two or three combined essential oils for testing inhibitory activity against *A. flavus* are presented in Table 1. This work demonstrated that cinnamon/oregano, cinnamon/lemongrass and oregano/lemongrass EOs have additive antifungal activity (fractional inhibitory concentration index (FICI) = 0.7500). On the other hand, the cinnamon-oregano-lemongrass combination shows synergistic activity (FICI = 0.4375). We also found that these three EOs have synergistic effects when cinnamon, oregano and lemongrass EOs are combined at a volume ratio of 1:5:48. The minimum inhibitory concentration of cinnamon, oregano and lemongrass oils in composite essential oils (CEOs) decreased 8-, 16- and 4-fold, respectively, when compared with individual EOs.

### 2.4. Inhibitory Effect of Cinnamon, Oregano and Lemongrass (COL)-CEO against AFB1 Biosynthesis at the Transcriptional Level

**Figure 3:**
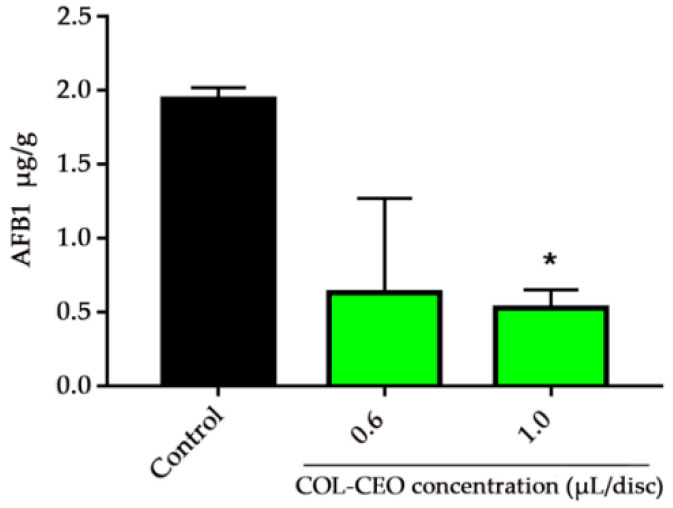
Effect of COL-CEO on AFB1 production. Asterisks indicate significant differences from the control group (*p* < 0.05). AFB1 production was measured as μg/g of mycelia. AFB1: aflatoxin B1; COL-CEO: composite essential oil of cinnamon, oregano and lemongrass.

The AFB1 production of fungal mycelia treated with different concentrations of cinnamon, oregano and lemongrass (COL)-CEO was investigated. The results showed that 0.6 μL/disc and 1.0 μL/disc of COL-CEO treatments inhibited AFB1 by 67.53% and 72.68%, respectively (Figure 3). It was evident that the inhibitory effect became more apparent with an increase in the concentration of COL-CEO.

**Figure 4:**
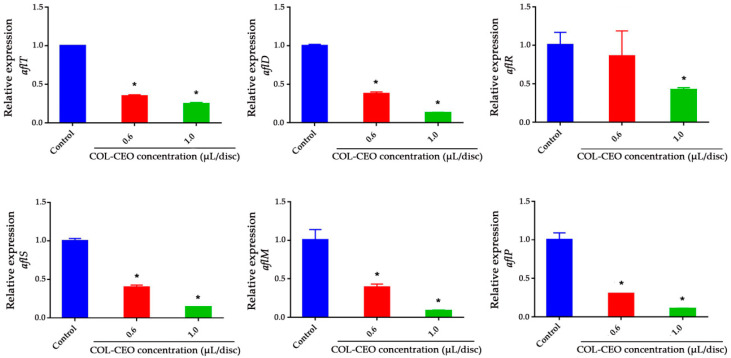
Expression levels of six AFB1 biosynthetic genes from *A. flavus* cultured at different COL-CEO concentrations for six days. *β*-tubulin was used for the standardization of gene expression. Asterisks indicate significant differences from the control group (*p* < 0.05). AFB1: aflatoxin B1; COL-CEO: composite essential oils of cinnamon, oregano and lemongrass.

To better understand the inhibitory effect of COL-CEO on AFB1 production, six genes involved in the aflatoxin biosynthetic pathway were selected for expression analysis by RT-qPCR. Varying degrees of relative expression patterns of these genes were observed relative to the reference gene *β*-tubulin. The expression of *aflT*, *aflD*, *aflP*, *aflM* and *aflS* genes was inhibited dramatically at the COL-CEO concentration of 0.6 μL/disc (*p* < 0.05) (Figure 4). In contrast to other genes, *aflR* was not significantly affected by COL-CEO treatment at 0.6 μL/disc (14.63%). An increase in the concentration of COL-CEO resulted in significant downregulation of the *aflR* gene at 1.0 μL/disc (58.04%). These results demonstrate that COL-CEO could modulate the expression of mycotoxin genes in the AFB1 biosynthetic pathway to inhibit AFB1 production in *A. flavus*.

### 2.5. Chemical Characterization of EOs

The chemical compounds in EOs identified by gas chromatography-mass spectrometry (GC-MS) analyses are listed in Table 2. A total of 14, 8 and 9 different components were detected from cinnamon, oregano and lemongrass EOs, respectively. These totals represented 99.09%, 98.99% and 98.58% of the total components of cinnamon, oregano and lemongrass EOs, respectively. Cinnamon EO was mainly composed of cinnamaldehyde (89.33%), (E)-2-methoxycinnamaldehyde (4.66%) and carveol (2.20%). In oregano EO, only two main components, carvacrol (84.96%) and thymol (13.26%), were identified. Limonene (5.83%), (Z)-citral (43.66%) and (E)-citral (43.55%) were the main components of lemongrass EO. GC-MS also identified 11 compounds representing 99.57% of the COL-CEO, which mainly consisted of (Z)-citral (33.44%), (E)-citral (32.88%) and carvacrol (19.84%), followed by limonene (4.29%) and cinnamaldehyde (3.76%). The chemical compositions of cinnamon, oregano and lemongrass EOs have been reported earlier. Although the proportion of each component differed from previous results, the main chemical composition was the same as our results [42,43].

**Figure 5:**
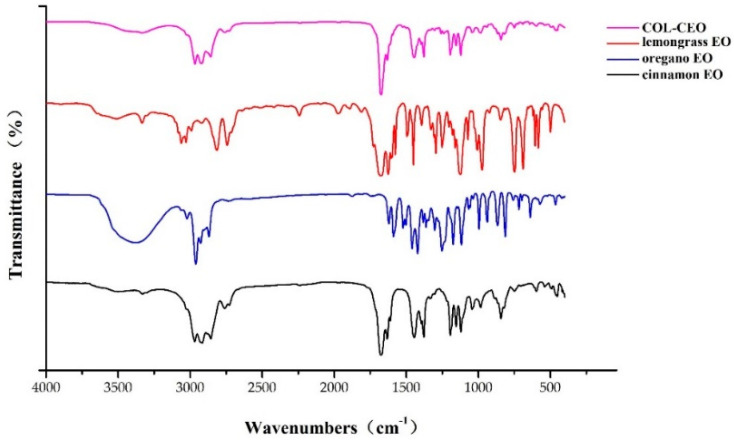
FTIR spectra of cinnamon EO, oregano EO, lemongrass EO, and COL-CEO. FTIR: Fourier transform infrared spectroscopy; EO: essential oil; COL-CEO: composite essential oils of cinnamon, oregano and lemongrass.

The Fourier transform infrared spectroscopy (FTIR) results spectra of cinnamon, oregano, lemongrass EOs and COL-CEO are presented in Figure 5. The characteristic differences in the absorption peaks of the EOs of these compounds indicated variations in their chemical constituents. The broad band at 3600 cm^−1^ and 3200 cm^−1^ was attributed to the fact that the O–H stretching band has the highest stretching strength in the oregano EO spectrum, relative to other EOs. The strong bands at 2916 cm^−1^ (cinnamon EO), 2961 cm^−1^ (oregano) and 2963 cm^−1^ (COL-CEO) indicate C–H stretching for an alkane. This band was not obvious in the lemongrass EO spectrum. The strong bands at 1674 cm^−1^ (cinnamon, lemongrass EOs and COL-CEO) revealed the presence of a carbonyl group C = O bond for aldehyde. This band was absent in the oregano EO spectrum. In the lemongrass EO spectrum, the peak at 1450 cm^−1^ represents a strong methyl band, whereas the band at 748 cm^−1^ (methyl rocking vibration) is indicative of a long-chain linear aliphatic structure. In addition, strong peaks at 841 cm^−1^ were attributed to the C–H vibration absorption of benzene rings. Overall, above results indicated that the major types of components in the essential oils of these compounds included aldehydes, alcohols, esters and phenols.

### 2.6. Antifungal Activity of COL-CEO against A. flavus on Maize Grains

**Figure 6:**
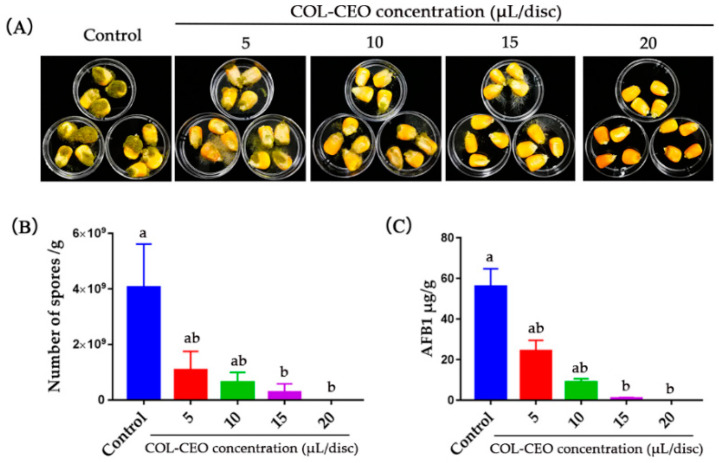
Effects of COL-CEO against *A. flavus* on maize kernels. (**A**) Representative photographs of maize samples treated with COL-CEO for 10 days; (**B**) Spore production of *A. flavus* on kernel surfaces in control and treatment groups. (**C**) AFB1 production of maize kernels treated with COL-CEO. AFB1 production was measured as μg/g of kernels. AFB1: aflatoxin B1; COL-CEO: composite essential oils of cinnamon, oregano and lemongrass. Each value is the mean of three replicates (*n* = 3) ± standard deviation (SD). Kruskal–Wallis test was applied for non-parametric analysis of variance, and means for each concentration were separated by uncorrected Dunn’s test. Values with different lower-case letters were significantly different (*p* < 0.05).

To further investigate the antifungal ability of COL-CEO on *A. flavus* in maize grains, kernels inoculated with *A. flavus* 3357 were exposed to vapors of 0, 5, 10, 15 and 20 μL/disc COL-CEO for 10 days. As shown in Figure 6A, COL-CEO could suppress *A. flavus* growth in stored maize grains in a dose-dependent manner. An increase in the concentration of COL-CEO decreased fungal growth on the kernels. Fumigation with COL-CEO at a concentration of 20 μL/disc completely protected maize grains from infection after 10 days of incubation (Figure 6B). Similarly, AFB1 accumulation was significantly reduced (Figure 6C). The dates demonstrated that COL-CEO is a potentially effective antifungal agent for controlling *A. flavus* growth in stored maize grains.

## 3. Discussion

In this study, the potency of essential oils in inhibiting mycelial growth and aflatoxin production in *A. flavus* was investigated. Essential oils can effectively control fungal contamination in food, with advantages such as being eco-friendly, along with high efficiency and low drug resistance [44]. Eleven EOs that showed high antifungal activity in previous studies were investigated for their antifungal activity against *A. flavus* NRRL 3357, a dominant AFB1-producing strain. Among these, mugwort [45], peppermint [46] and rosemary EOs [47,48] were previously proposed as potential preservatives, with the ability to extend the shelf life of food [49,50,51]. However, in this study, these three EOs had no visible antifungal effect on *A. flavus* NRRL 3357. Moreover, in the current work, the antifungal effects of cinnamon, oregano and lemongrass EOs were found to be the highest among all EOs tested. In particular, cinnamon EO showed remarkable antifungal activity. Previous studies have reported that cinnamon EO can inhibit fungal growth and mycotoxin production by *A. flavus* [37,52], with its major antifungal ingredient being cinnamaldehyde. In our study, cinnamon EO was found to have about 89.33% cinnamaldehyde.

Many studies have reported that the antifungal activity of EOs can be increased by combining two or more essential oils to create a synergistic antifungal effect of EO vapors. For example, an oregano and thyme EO mixture was shown to have synergistic effects against molds (*A. niger*, *A. flavus*, *A. parasiticus*, and *P. chrysogenum*) [53]. The combination of cinnamon bark, citronella and May Chang EO vapors had synergistic inhibitory effects on *Penicillium corylophilum* [39]. The essential oil from cinnamon has shown remarkable antimicrobial activity. It was also found that cinnamon and oregano EO had varying effects on the growth of *A. flavus* [53]. Marín [41] mentioned that a mixture of cinnamon, lemongrass and oregano EOs showed lower antifungal ability. However, we found that cinnamon, oregano and lemongrass CEO (COL-CEO) with a volume ratio of 1:5:48 (*v*:*v*:*v*) showed a synergistic effect against *A. flavus*. The MIC of COL-CEO was reduced when compared with individual EOs.

The major antifungal components in plant-derived essential oils have been intensively investigated, and can be arranged as follows: phenols—cinnamic aldehyde—alcohols—aldehydes = ketones—ethers—hydrocarbons [54]. Among these, aldehydes, phenols and alcohols are the main antifungal ingredients [42,55]. In this study, although the cinnamaldehyde in COL-CEO decreased to 3.76% when compared with cinnamon oil, other active ingredients such as (Z)-citral, (E)-citral [43], limonene [56] and carvacrol [57] were increased. The combined formulation of these alcohols and aldehydes may result in synergistic effects and contribute to the observed antifungal activity of COL-CEO against *A. flavus*.

Inhibition of aflatoxin production by essential oils is related to the downregulation of aflatoxin biosynthesis genes. Previous investigations have shown that the genes for aflatoxin biosynthesis are clustered in the genome of *Aspergillus* [58,59]. Twenty-five genes have been identified from the 70 kb gene cluster in *A. flavus* [60,61]. Of these, two regulatory genes, *aflR* and *aflS*, and four structural genes, *aflT*, *aflD*, *aflM* and *aflP*, were investigated in the present study. *aflT* (*pksA*) encodes polyketide synthase and *aflD* (*nor-1*) encodes reductase involved in the early stage of the aflatoxin synthesis pathway. *aflM* (*ver-1*) encodes dehydrogenase, involved in the middle of the pathway. *aflP* (*omtA*) encodes O-methyltransferase A, implicated in the late stage. In the present study, these genes were found to be downregulated by COL-CEO, which was consistent with the observation that aflatoxin B1 production was reduced in all samples grown under COL-CEO vapor. Furthermore, downregulation of these genes may be in response to reduced oxidative stress caused by CEO. Previous studies have shown that oxidative stress due to the accumulation of intracellular reactive oxygen species can enhance the production of AFB1 in *A. flavus* and *A. parasiticus* [62,63]. Moreover, several investigations have also indicated that antioxidants (such as citral, cinnamaldehyde and eugenol) can significantly reduce aflatoxin production, whereas oxidants enhance aflatoxin production [64,65,66,67,68].

A maize grains infection assay demonstrated that COL-CEO has high inhibitory activity towards *A. flavus* on maize grains. Cinnamon and oregano have a pungent odor and are difficult to accept in food, whereas lemongrass EO has an aromatic odor [69]. These three essential oils, when combined, maximize consumer satisfaction. The application of COL-CEO may be a useful strategy for future grain and food packaging and storage.

## 4. Conclusions

In this study, the antifungal effect of 11 EOs on *A. flavus* NRRL 3357 was investigated. Cinnamon, oregano and lemongrass EOs in the vapor phase were found to have the optimal antifungal effects. Furthermore, the three EOs had a synergistic effect when combined at a volume ratio of 1:5:48, respectively, as indicated by FICI values. Aldehydes and alcohols were identified by FTIR and GC-MS as the main components in COL-CEO. The COL-CEO showed remarkable inhibitory activity against *A. flavus* in terms of both mycelial growth and aflatoxin production. Downregulation of *aflT*, *aflD*, *aflS*, *aflM* and *aflP* genes in aflatoxin biosynthesis pathway upon COL-CEO treatment contributed to lower aflatoxin production. Most importantly, COL-CEO had an excellent inhibitory effect upon colonization of *A. flavus* on maize grains. These results indicated that COL-CEO is a potential natural antifungal agent, which can be used for the storage of maize and other grains.

## 5. Materials and Methods

### 5.1. Essential Oils and Fungal Strain

The essential oils (EOs) used in this study were cinnamon, oregano, lemongrass, clove, citronella, basil, thyme, *Litsea cubeba*, peppermint, mugwort and rosemary (Jiangxi Xuesong Natural Medicinal Oil Co. Ltd., Jiangxi, China). These EOs were stored at room temperature in the dark before use. All the EOs were used directly without any dilution.

An aflatoxin-producing strain, *A. flavus* NRRL 3357, was used to evaluate the antifungal effect of essential oils. The strain was cultivated on potato dextrose agar (PDA, Difco, BD Co. Ltd., Sparks, MD, USA) plates and incubated at 28 ± 2 °C for seven days. Fresh conidia were then harvested and diluted with sterilized water to a concentration of 10^6^ spores/mL. Spore count was estimated using a hemocytometer. The conidial suspension was used as inoculum for all cultivation states.

### 5.2. Antifungal Activity of EOs on Growth of A. flavus

We used vapor methods [70], with some modifications, to determine the antifungal activity of the EOs on *A. flavus* mycelial growth. Petri dishes (90 mm × 20 mm) were filled with 20 mL of PDA medium, and conidial suspension (1 × 10^4^ spores) was seeded centrally onto the PDA plates. Autoclaved filter discs (70 mm in diameter, Whatman No. 2) containing 1, 5, 10, 25 and 50 μL of EOs were placed at the center of each Petri dish lid. Blank filter discs were used as control samples. All Petri dishes were sealed with Parafilm^®^ tapes and incubated at 28 ± 2 °C in an inverted position. The diameters of the colonies in the plates were recorded after 7-day culture. Three replicates were performed for each treatment. To facilitate comparisons between EOs, this study expressed the unit of concentration as microliter per disc (μL/disc) [71], which can also be expressed as microgram per milliliter (μg/mL) (Table S1).

### 5.3. Determination of Minimum Inhibitory Concentration (MIC)

MIC analysis was applied to determine the antifungal activities of cinnamon, oregano and lemongrass EOs under vapor conditions. Different concentrations were chosen to determine the MIC of the three EOs. Concentrations of 0.10, 0.15, 0.20 and 0.25 μL/disc were used for cinnamon EO. For oregano EO, concentrations of 1.00, 1.50, 2.00 and 2.50 μL/disc were used, whereas concentrations of 3.00, 4.00, 5.00 and 6.00 μL/disc were used for lemongrass EO. The cultures were incubated at 28 ± 2 °C for seven days. Each treatment was carried out thrice. MIC was defined as the lowest EO concentration that completely inhibited visible growth of *A. flavus* [72,73].

### 5.4. Combinatorial Assays

**Figure 7:**
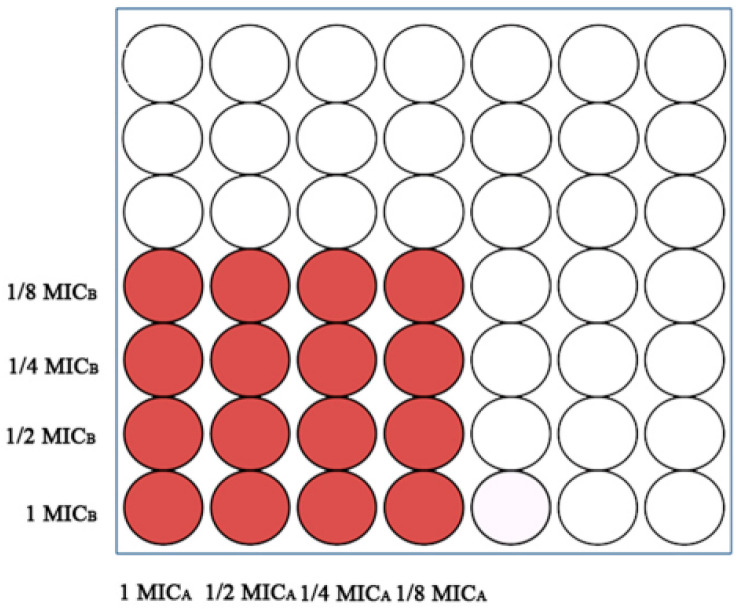
Schematic diagram of checkerboard assay. A represents one essential oil and B represents another essential oil. The white wells were empty.

Combinations of two or three undiluted EOs (Figure 7) were evaluated for synergistic inhibitory activity against *A. flavus* using a checkerboard assay [72]. Concentrations of 1 MIC to 1/8 MIC of cinnamon EO, oregano EO and lemongrass EO were added to sterile filter discs, which were then placed in the center of the lid of Petri dishes. The plates were sealed with Parafilm^®^ tapes and incubated at 28 ± 2 °C for seven days. When there was no *A. flavus* growth, the fractional inhibitory concentration (FIC) and fractional inhibitory concentration index (FICI) were calculated to confirm and verify the occurrence of synergistic antifungal activity. According to European Committee for Antimicrobial Susceptibility Testing (EUCAST) guidelines [74], a combination with a FICI value ≤ 0.5 was considered to have a synergistic effect, an additive effect if 0.5 < FICI value ≤ 1, an indifferent effect when 1 < FICI value < 2 and an antagonistic effect when FICI value ≥ 2.

For each replicate, FICI values were calculated using the following formula [75]:

FICI = FIC (A) + FIC (B)

where A and B represent two different essential oils. FIC (A) = MIC of CEO/MIC for essential oil A alone and FIC (B) = MIC of CEO/MIC for essential oil B alone.

To measure the synergistic activity of a combination of three combined EOs against *A. flavus*, two-fold serial dilutions from 1 MIC to 1/8 MIC of cinnamon, oregano and lemongrass EOs were prepared. Each of the cinnamon (1, 1/2, 1/4 and 1/8 MIC) and oregano EOs (1, 1/2, 1/4 and 1/8 MIC) were first mixed with each other. The resulting EO mixtures were then added to the lemongrass EOs (1, 1/2, 1/4 and 1/8 MIC). The resulting 96 combinations of cinnamon, oregano and lemongrass EOs were tested for their inhibitory activity. The Petri dishes were covered with a lid and incubated at 28 ± 2 °C for seven days, following which the diameters of the colonies in the plates were measured. Synergistic antifungal effects of various EO combinations were confirmed by calculating their FIC and FICI values as per the equations above.

### 5.5. RNA Extraction and RT-qPCR Analysis

To investigate transcriptional inhibition of AFB1 biosynthesis by CEO, conidial suspension (1 × 10^4^ spores) was seeded onto the PDA plate and incubated at 28 ± 2 °C. Mycelia of *A. flavus* treated with 0.6 μL and 1.0 μL/disc of CEO for six days were analyzed and compared with the control, consisting of a sample without CEO treatment. Total RNA was isolated using RNAiso Plus reagent (TaKaRa Co. Ltd., Otsu, Shiga, Japan) according to the manufacturer’s instructions. cDNA was synthesized from 1 μL of total RNA by reverse transcription, using a TransScript One-Step gDNA Removal and cDNA Synthesis SuperMix (Transgen, Co. Ltd., Beijing, China). The reaction mixture was first incubated for 5 min at 65 °C and the PCR program was at 25 °C for 10 min, 42 °C for 15 min, 85 °C for 5 s and 40 °C for 5 s.

The *A. flavus* NRRL 3357 genome database sequence was used to design primers for genes in the 75 kb aflatoxin biosynthesis gene cluster. Six genes involved in the aflatoxin biosynthetic pathway, *aflT*, *aflD*, *aflR*, *aflS*, *aflM* and *aflP*, were selected for RT-qPCR analysis (Table 3). Primers were synthesized by General Biosystems (Chuzhou, China). Real-time quantitative RT-PCR (PikoReal 96 Real-Time PCR System, Ventaa, Finland) was carried out using the TB Green^®^ Premix Ex Taq^TM^ II (TaKaRa Co. Ltd., Otsu, Shiga, Japan), in a final volume of 20 μL, consisting of 10 μL TB Green Premix Ex Taq II (2x), 0.5 μL of each primer (10 μM) and 1 μL cDNA. The qPCR program included an initial denaturation at 95 °C for 30 s, followed by a 2-step PCR, 40 cycles of 95 °C for 5 s and 60 °C for 30 s. The *β-*tubulin gene was used as an endogenous control, with three biological replicates assessed for each sample. Relative expression levels were calculated using the comparative CT (2^−ΔΔ*C*T^) method.

### 5.6. Gas Chromatography-Mass Spectrometry (GC-MS) Analysis of the EOs

The chemical composition of EOs was evaluated by GC-MS (Agilent Technologies 7890B/7000, Palo Alto, CA, USA) equipped with a DB-5ms Ultra Inert column (60 m × 0.25 mm × 0.25 μm). Ultrapure nitrogen was used as a carrier gas at a flow rate of 1.0 mL/min. EO (10 μL) was mixed with a dichloromethane solvent (990 μL). The injection volume was 0.1 μL with a split ratio of 20:1. The initial column temperature was held at 50 °C for 5 min, programmed to 180 °C at a rate of 4 °C/min for 4 min, then 280 °C at a rate of 4 °C/min for 2 min and held at this temperature for 60 min. The mass spectra were taken at 70 eV. Mass range was from 40 to 500 amu. The percentage composition of the oil was computed from GC peak areas. Individual components were also identified by comparing their retention times and mass spectra with the NIST Mass Spectral Library (NIST 11) and NIST MS Search Program v2.0g.

### 5.7. Fourier Transform Infrared Spectroscopy (FTIR) Analysis of EOs

To monitor changes in the chemical groups of essential oils after mixing, samples (cinnamon, oregano, lemongrass EO and CEO) were evaluated using an FTIR spectrometer (Nicolette iS50, Thermofisher, Madison, WI, USA) equipped with an attenuated total reflectance (ATR) accessory. FTIR analysis was performed at a range of 40 to 4000 cm^−1^, with a resolution of 4 cm^−1^ for 64 scans.

### 5.8. CEO Vapors against A. flavus on Maize Grains

A laboratory kernel infection assay (KIA) was performed using maize grains sourced from a local field. Undamaged maize kernels were sterilized with 1% sodium hypochlorite, washed thrice with sterile water and vacuum dried in an oven at 55 ± 2 °C for 30 min. The kernels were then immersed in *A. flavus* spore suspension (1 × 10^6^ spores/mL) for 5 min. Four kernels were placed in individual 35 mm Petri dishes without a lid, and three such small dishes were then placed in a large Petri dish (90 mm × 20 mm). For the CEO vapor group, 5, 10, 15 and 20 μL of CEO were spotted on a filter paper and placed on the small dishes. The large Petri dishes were sealed with Parafilm^®^ tapes. An untreated sample served as the control, and each test was repeated three times. High humidity (>95% relative humidity (RH)) was maintained by adding double-distilled water to the large dishes. Spore counts and AFB1 production in maize grains were determined following a 10-day culture at 28 °C. Spores were harvested by washing maize kernels with 0.01% Triton X-100 and spots were counted with a hemacytometer. Maize kernels were then dried for two days in an oven at 60 ± 2 °C and weighed. Spore production was measured as the number of spores/g of maize grains. For aflatoxin analysis, the whole kernels covered with mycelia were used. Aflatoxin B1 production was measured as μg/g of maize grains.

### 5.9. Aflatoxin Analysis

Quantitative comparison of the production of aflatoxin B1 among various treatments was conducted as previously described [76,77], with few modifications. For aflatoxin analysis of mycelia treated with essential oil, the conidial suspension was seeded centrally onto sterile cellophane sheets placed over PDA plates and incubated at 28 ± 2 °C for seven days. The fungal biomass was scraped from the plates and disrupted using a grinder after weighing. The samples were added to 10 mL of 100% methanol and incubated in a shaker (200 rpm) at room temperature for 2 h. For aflatoxin analysis of infected maize grains, the maize kernels were dried for two days in a 60 ± 2 °C oven and ground to a powder. The weighed powder from 12 maize grains together formed one group that was added to 10 mL of methanol and incubated in a shaker (200 rpm) at room temperature for 2 h. The supernatant was then collected by centrifugation at 3000× *g* for 10 min at room temperature and filtered through a 0.22 μm syringe filter (Alltech).

Each sample was analyzed by Waters 600 Controller HPLC equipped with a fluorescence detector (Waters 2475 Multi λ Fluorescence Detector, Milford, MA, USA). The chromatogram was recorded at 365 nm excitation and 465 nm emission wavelength using a reverse-phase column Luna 3u C18 (2), 150 mm × 4.6 mm × 3 μm (Phenomenex, Torrance, CA, USA), and an isocratic mobile phase with a flow rate of 0.6 mL min^−1^, consisting of 55% water and 45% methanol. Three replicates were analyzed for each concentration. The limit of detection (LOD) and the limit of quantification (LOQ) of HPLC for AFB1 were 1.5~2.0 ppb and 3.0~4.0 ppb, respectively.

### 5.10. Statistical Analysis

All experimental results were reported as mean ± standard deviation (SD). The Shapiro–Wilk and Brown–Forsythe tests were used to check for normality and homoscedasticity, respectively. Data with these conditions were evaluated by analysis of variance (ANOVA). Data that did not meet normality and homoscedasticity criteria were analyzed by the non-parametric Kruskal–Wallis test. Statistical analyses were performed using GraphPad Prism 8.0 software (GraphPad Software, San Diego, CA, USA). The significance level was set at *p <* 0.05.

## Figures and Tables

**Table 1:** Combinations of two or three essential oils tested for inhibitory activity against *A. flavus.*

Concentration of EO (μL/disc)			Total	FIC			FICI	Antifungal Activity
Cinnamon	Oregano	Lemongrass		Cinnamon	Oregano	Lemongrass		
0.1250	0.6250	-	0.750	0.5000	0.2500	-	0.7500	Additive
0.1250	-	1.5000	1.625	0.5000	-	0.2500	0.7500	Additive
-	1.2500	1.5000	2.750	-	0.5000	0.2500	0.7500	Additive
0.0313	0.1563	1.5000	1.6876	0.1250	0.0625	0.2500	0.4375	Synergistic

EO: essential oil; FIC: fractional inhibitory concentration; FICI: fractional inhibitory concentration index.

**Table 2:** Chemical compounds of individual and composite essential oils.

Peak No.	Retention Time, Minutes	Name	Cinnamon%	Oregano%	Lemongrass%	COL-CEO%
1	15.607	6,6-Trimethyl-(1 theta)-bicyclo [3.1.1]hept-2-en	0.08	0.03	ND	ND
2	16.637	Benzaldehyde	1.04	ND	ND	ND
3	18.883	Limonene	ND	0.02	5.83	4.29
4	22.785	(S)-Cis-verbenol	0.09	ND	0.89	0.63
5	23.026	Phenylpropyl aldehyde	0.19	ND	ND	ND
6	24.583	Carveol	2.20	ND	1.70	1.18
7	25.062	(Z)-Citral	0.43	ND	43.66	33.44
8	25.45	Phenethyl acetate	0.09	ND	ND	ND
9	25.835	(E)-Citral	ND	ND	43.55	32.88
10	26.271	Cinnamaldehyde	89.33	0.03	0.1	3.76
11	26.349	Thymol	ND	13.26	ND	1.78
12	26.717	Carvacrol	ND	84.96	ND	19.84
13	27.428	2,3-Epoxygeranial	0.19	0.02	1.30	0.76
14	27.822	Hexa-1,3-dien-1-ylbenzene	0.15	ND	ND	ND
15	28.479	(2,2,6-Trimethylbicyclo [4.1.0]hept-1-yl)methanol	0.17	0.08	1.49	0.94
16	28.711	2-Pentadec-12-ynoxyoxane	ND	ND	0.06	0.07
17	28.979	2-Vinylnaphthalene	0.17	ND	ND	ND
18	32.786	(E)-2-Methoxycinnamaldehyde	4.66	ND	ND	ND
19	35.951	4-Phenylbenzaldehyde	0.30	ND	ND	ND
20	46.199	3,3,5,5-Tetramethyl-8-(3-methylbutyl)-6,7-dihydro-2H-s-indacen-1-one	ND	0.59	ND	ND
total			99.09	98.99	98.58	99.57

ND: compounds not detected. COL-CEO: composite essential oils of cinnamon, oregano and lemongrass.

**Table 3:** Primers used in RT-qPCR analysis.

Gene	Primer Sequence (5’–3’)	Target Fragment Length (bp)
*β-*tubulin	F: GACACCGTTGTTGAGCCCTA	135
	R: GTCACCGTAAGAGGGGTTGG	
*aflT*	F: CCATTCGCAACCAATCACC	105
	R: GCCAATCGACATGATCACGC	
*aflD*	F: GCCATCACGGTCAAGAGAAG	106
	R: GCCTTCAGCGACGGTTAGTC	
*aflR*	F: CCACTACCACCGTTTCAGGC	88
	R: ATGCCAGCACCTTGAGAACG	
*aflS*	F: CGCTCACTCGGATGAACTGG	109
	R: CCAGACTCGGCCTTAGCTTC	
*aflM*	F: ATCCTGAGGCAACTGCGAAG	100
	R: TCACGCAAGCAGTGTTAGAGC	
*aflP*	F: AACTCCCTCCTCCACCGAAC	109
	R: CGATTGTGTCGAGTGATGTGG	

## Supplementary Materials

The following are available online at https://www.mdpi.com/2072-6651/12/9/562/s1, Table S1: Conversion of concentration units of essential oils.
